# Socket Preservation Using Dense Polytetrafluoroethylene Membranes and Platelet-Rich Plasma

**DOI:** 10.7759/cureus.72265

**Published:** 2024-10-24

**Authors:** Ralitsa V Yotsova

**Affiliations:** 1 Department of Oral Surgery, Medical University of Varna, Varna, BGR

**Keywords:** dense polytetrafluoroethylene membrane, platelet-rich plasma, post-extraction ridge resorption, ridge preservation, socket preservation

## Abstract

Socket preservation (SP) is a method aimed at reducing the post-extraction resorption of the alveolar crest and promoting bone deposition in the socket. It involves procedures such as atraumatic tooth extraction, guided regeneration with barrier membranes and bone substitutes, socket sealing, and immediate implant placement. This research aims to evaluate the influence of the combination of dense polytetrafluoroethylene (d-PTFE) membranes and platelet-rich plasma (PRP) on the vertical post-extraction resorption at the premolar and molar sites. Forty participants, aged 18-65 years, who needed extraction of a premolar or molar were enrolled and randomly assigned to an experimental group (SP with d-PTFE membrane and PRP) or a control group (spontaneous socket healing). The results demonstrated that SP with non-porous PTFE membranes and PRP reduced vertical bone resorption at the premolar and molar sites. Data analysis suggested that the buccal plate width influences the amount of its vertical resorption, but not as much as the management of the post-extraction socket (SP versus spontaneous socket healing). Further longitudinal randomized controlled trials are necessary to clarify which factors influence post-extraction ridge resorption and evaluate the success of different SP techniques.

## Introduction

The alveolar ridge is a structure that undergoes significant resorption after tooth loss. Post-extraction healing is characterized by intrinsic alterations leading to bone deposition in the socket and extrinsic alterations leading to dimensional reduction of the crest [1]. Post-extraction resorption can pose esthetic, functional, and prosthetic challenges, including difficulties in implant treatment. It is an irreversible process that leads to an alveolar width reduction of 2.6-4.6 mm and an alveolar height reduction of 0.4-3.9 mm [2].

Socket preservation (SP) or ridge preservation (which is the more broadly accepted term) is a method that aims to preserve the volume of the alveolar crest following tooth extraction [2,3]. SP involves various procedures, such as atraumatic tooth extraction, guided regeneration with barrier membranes and bone substitutes, socket sealing, and immediate implant placement [4].

Dense polytetrafluoroethylene (d-PTFE) membranes have recently gained popularity in bone regenerative therapy and SP. Since their surface is impervious to bacteria, they can be left exposed to the oral cavity without flap mobilization and soft tissue covering. Thus, the mucogingival junction and the depth of the vestibule remain unchanged. Membrane removal usually does not require a second surgery [5].

Autologous platelet concentrates (APCs) represent autologous blood products that enhance tissue healing and regeneration [6]. They stimulate osteoid formation and epithelialization of the socket due to the release of growth factors and cytokines [7].

Platelet-rich plasma (PRP) is a concentration of autologous platelets in a small volume of plasma obtained by centrifugation. It is a valuable source of bioactive factors [8]. PRP accelerates healing processes and improves hemostasis in bone and soft tissue defects. Due to its autogenous origin, there is no risk of infection transmission or a recipient reaction. PRP can increase bone formation rate up to 2.18 times [9]. It has been widely adopted in different medical fields, such as dermatology, traumatology, oral surgery, periodontology, and dental implantology [10-13].

This research aims to analyze the influence of d-PTFE membranes and PRP on the vertical post-extraction ridge resorption at the premolar and molar sites.

## Materials and methods

Study design and setting

This randomized controlled trial was conducted at the University Medical and Dental Centre, Medical University of Varna, Bulgaria, from June 2022 to April 2023.

Ethical approval (No.: 118/23 June 2022) was obtained from the Research Ethics Committee of the Medical University of Varna, Bulgaria, and the study was registered on ClinicalTrials.gov (registration protocol: NCT06621498). Written informed consent was obtained from all patients before the extraction.

Study subjects

**Figure 1:**
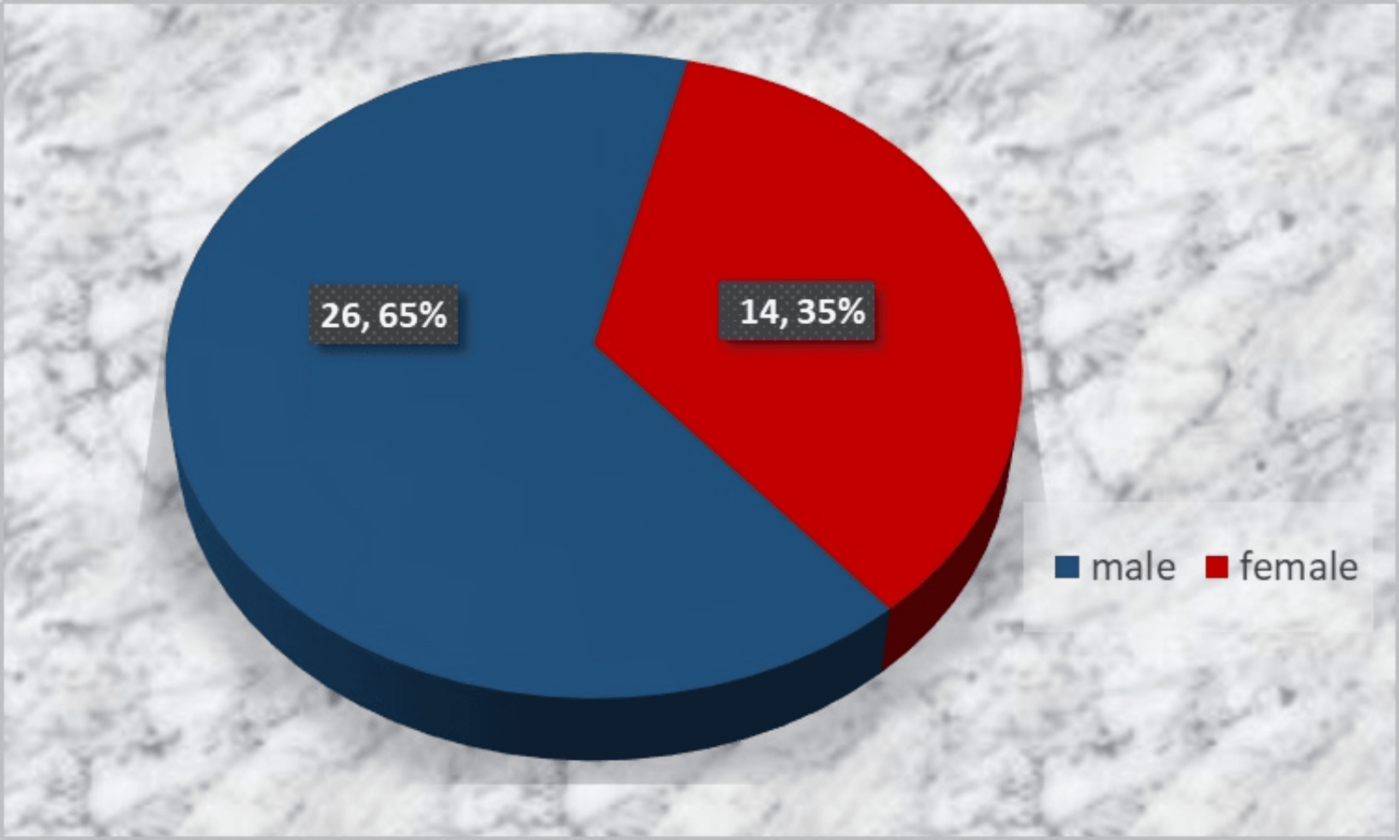
Gender distribution of the study subjects

**Figure 2:**
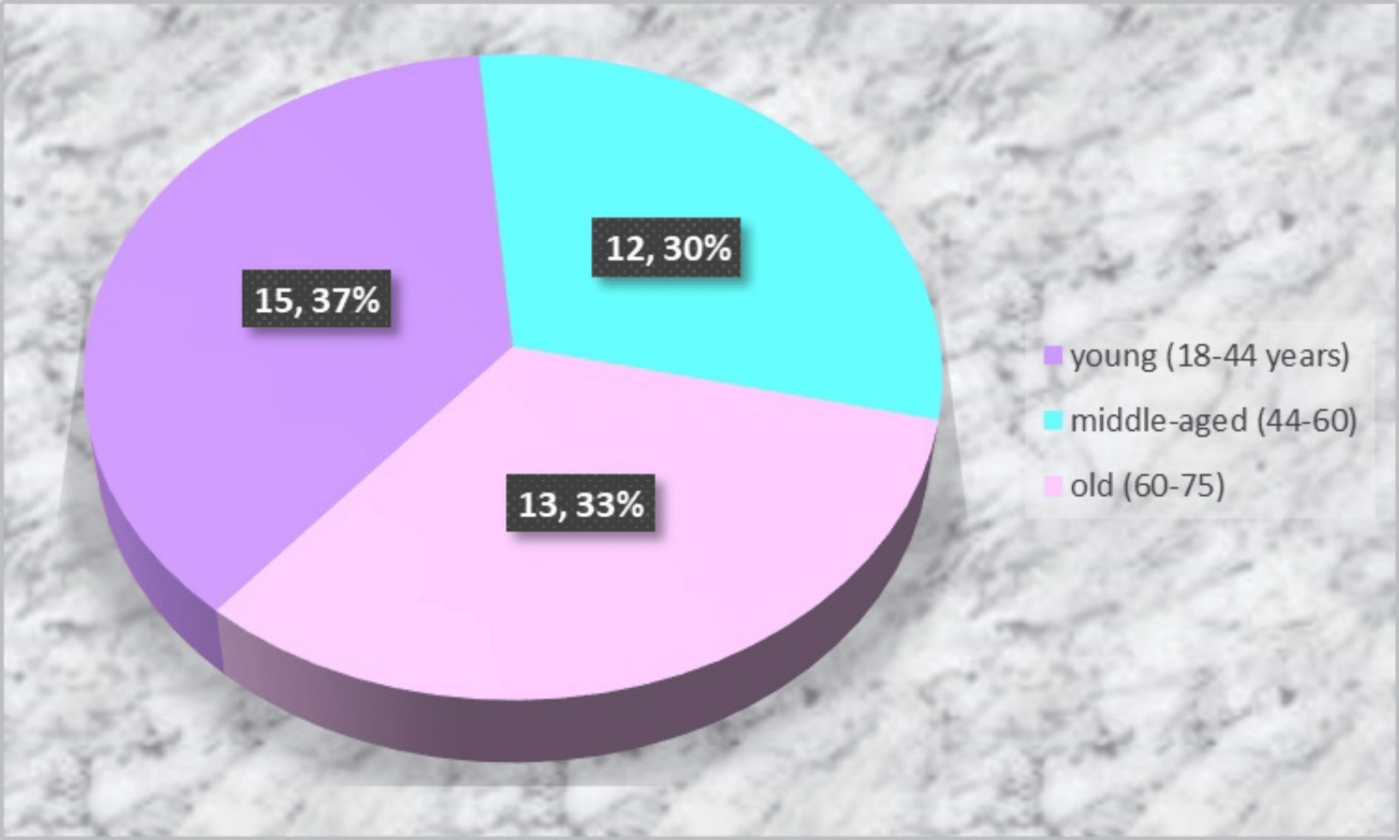
Age distribution of the study subjects

Forty patients who needed extraction of a premolar or molar were enrolled and randomly (lottery-based) assigned in equal numbers to the experimental group (SP with a d-PTFE membrane and PRP) or the control group (spontaneous socket healing), irrespective of the tooth type. Inclusion criteria were patients aged 18-65 years in good general health, patients who needed a premolar or molar extraction, and signed informed consent for participation in the study. Exclusion criteria were general (uncontrolled hypertension, uncontrolled metabolic disorders, acute infections, immunosuppressive therapy, chemotherapy or radiotherapy in the last six months, and so on) and local contraindications (local infection, inflammation, and neoplasm) for oral surgery procedures as well as lack of informed consent or patient's motivation to participate in the study. Figures 1, 2 display the distribution by age and gender.

Patient recruitment

Patient recruitment was performed after careful evaluation of the indications for treatment and the absence of general and local contraindications for oral surgery procedures.

Patient examination

Each participant underwent clinical and radiological examinations. The latter included a pre-operative X-ray (an orthopantomogram or a periapical X-ray) and two postoperative cone-beam computed tomography (CBCT) scans; the first CBCT scan was taken on the extraction day, and the second one was taken after three months. CBCT has been widely used for three-dimensional evaluation of the alveolar crest [14,15]. The measurements on the first CBCT scan included the height and width of the buccal plate (A0 and Aw) and the oral plate (B0 and Bw). Measurements were made on a paraxial section in the center of the socket (a vertical line drawn in the middle of the horizontal line connecting the mesial and distal osseous margins of the socket). Plate heights were measured by drawing two straight lines from the uppermost point of each bone plate to the uppermost point of the mandibular canal for teeth on the lower jaw and the lowermost point of the maxillary sinus for teeth on the upper jaw, respectively. These landmarks were selected as stable reference points instead of the bottom of the socket due to the rapid resorption of the bundle bone, which covers it inside. Plate widths were taken 3 mm apically from the uppermost points of the bone plates.

**Figure 3:**
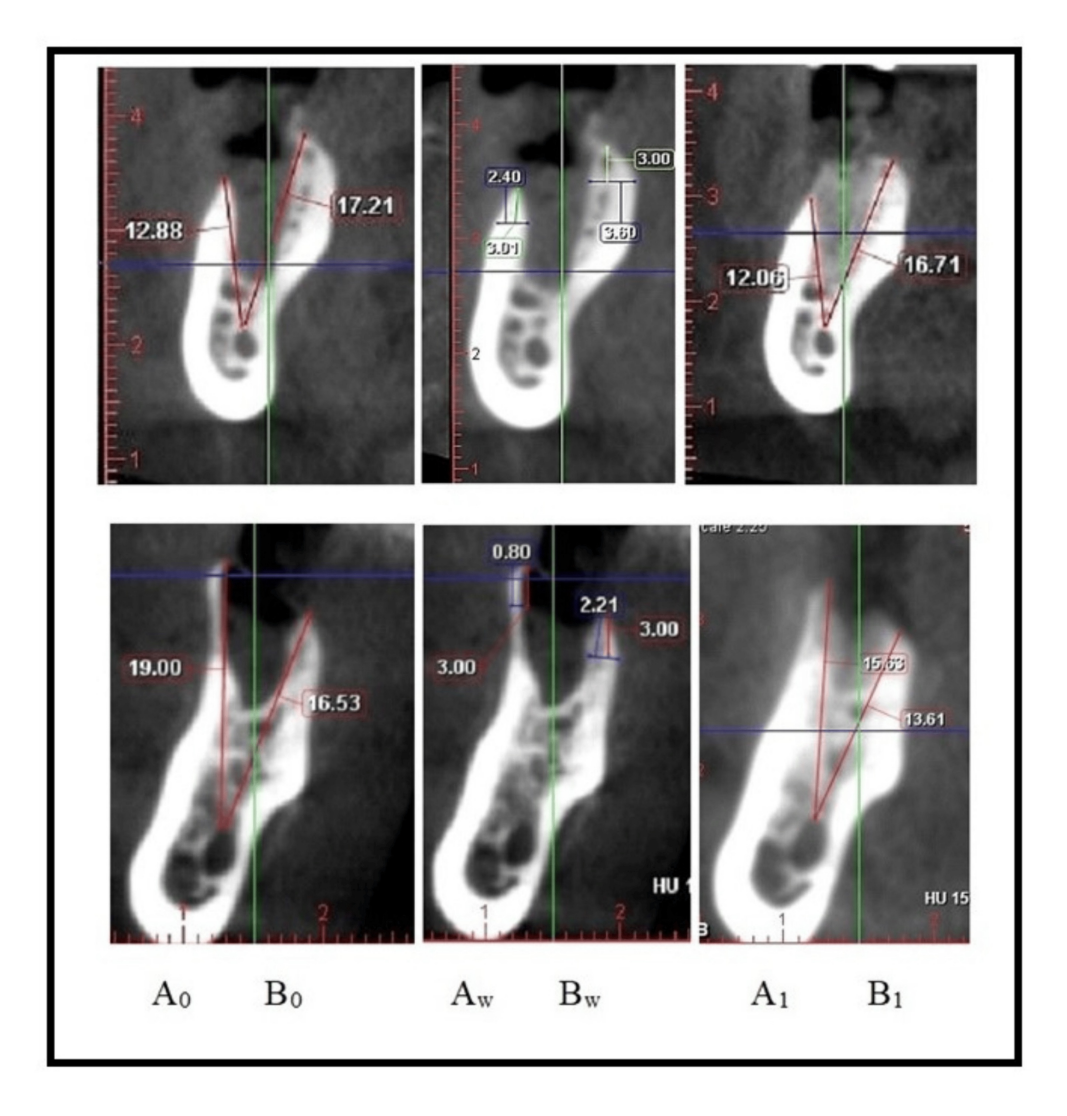
Cone-beam computed tomography images on the day of the extraction (left and middle) and three months after the surgery (right): paraxial slices A0: Buccal bone height on the day of extraction; B0: Palatal/lingual bone height on the day of extraction; Aw: Buccal bone width on the day of extraction; Bw: Palatal/lingual bone width on the day of extraction; A1: Buccal bone height three months after the extraction; B1: Palatal/lingual bone height three months after the extraction.

In the subsequent data analysis, the patients of each study group were divided into two subgroups, namely, Aw ≤ 2 mm and Aw > 2 mm, to evaluate the influence of the plate thickness on vertical bone resorption. In the case of preserved interradicular septa at the molar post-extraction sites, measurements were performed in both mesial and distal parts of the socket. The measurements on the second CBCT scan included the heights of the buccal (A1) and palatal/lingual plates (B1) after three months (Figure 3).

**Figure 4:**
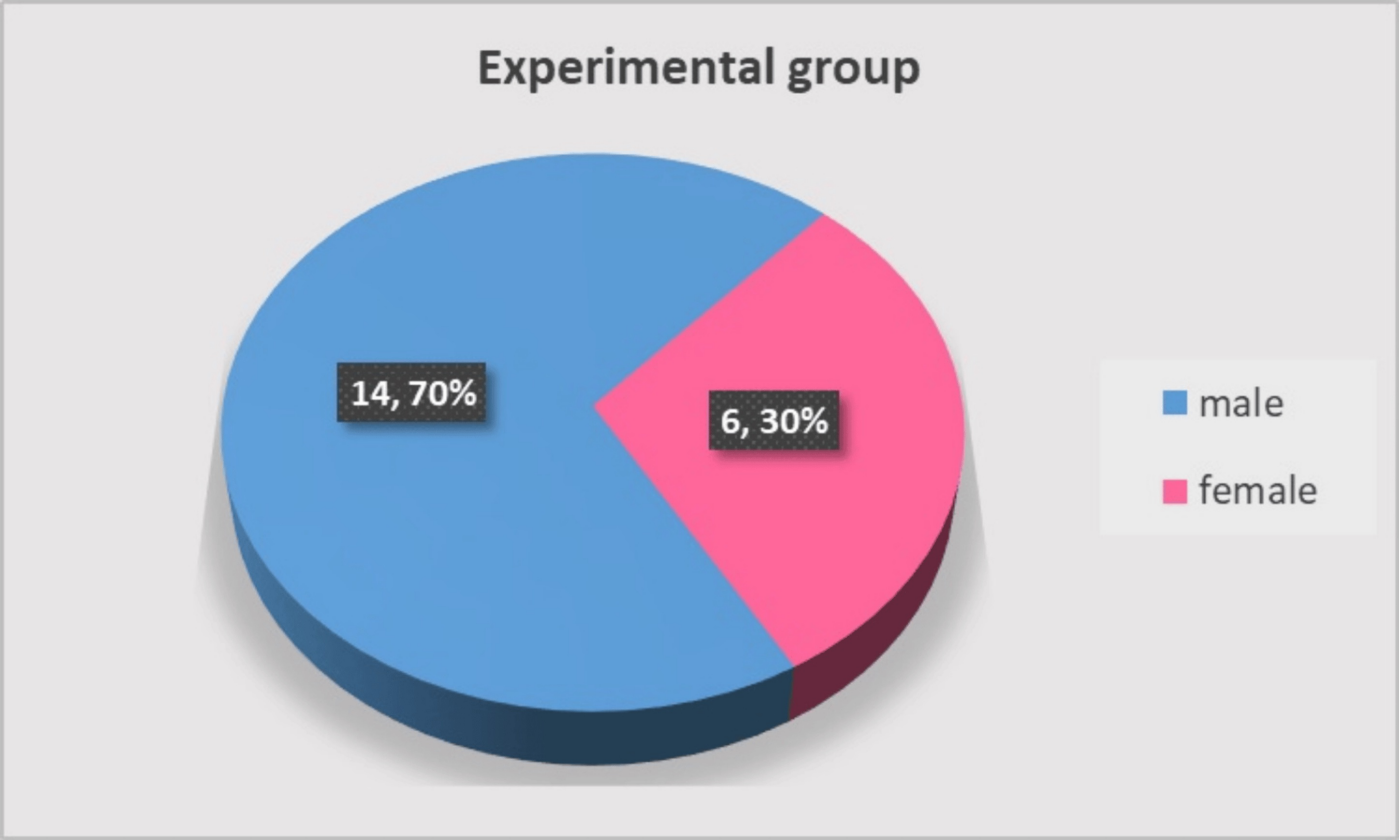
Gender distribution in the experimental group

The experimental group included 20 patients between 29 and 65 years, with the average age being 48.05 ±14.23 years. Regarding the gender distribution, 14 participants (70%) were male, and six (30%) were female, with no statistically significant gender difference (c2 = 3.20, p = 0.074), as shown in Figure 4. The extracted teeth in the group were nine premolars and 11 molars.

**Figure 5:**
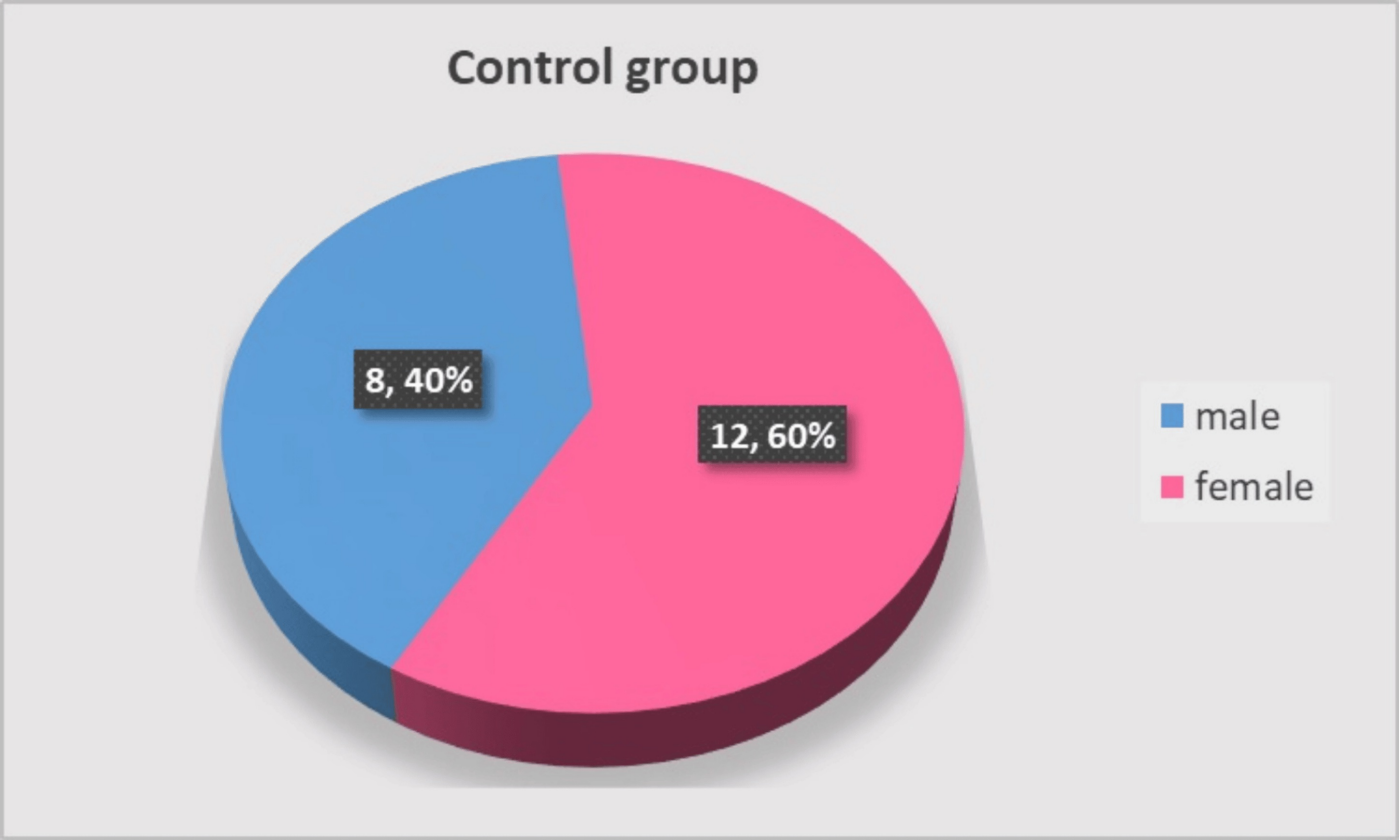
Gender distribution in the control group

The control group consisted of 20 participants aged 31 to 65 years. There was no significant difference in gender distribution: 12 participants (60%) were male, and eight (40%) were female (c2 = 0.80, p = 0.371), as shown in Figure 5. The extracted teeth in the group were eight premolars and 12 molars.

Protocol for PRP preparation

**Figure 6:**
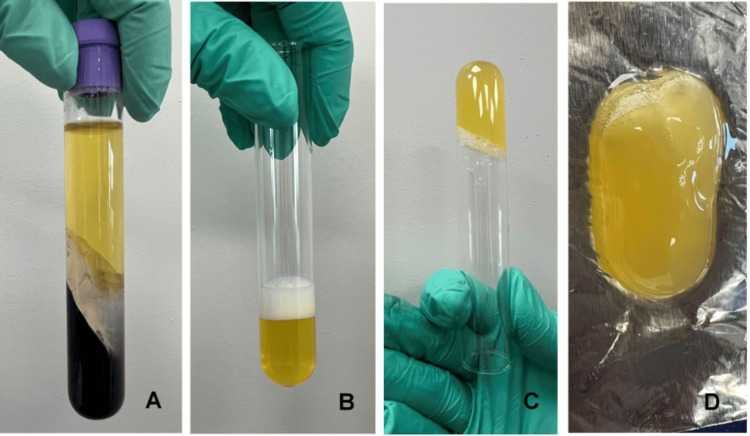
Platelet-rich plasma preparation (A) After the first spinning, (B) after the second spinning, (C) and (D) activated PRP. PRP: Platelet-rich plasma.

The methodology was first introduced by Ivanova et al. in 2021 [16] and adopted by clinicians and researchers in the Medical and Dental Centre at the Medical University of Varna, Bulgaria. Eight milliliters of blood were collected from the patient’s cephalic or median cubital vein. The blood was transferred to a vacutainer, containing an anticoagulant and a separating gel, and then centrifuged at 3500 rpm/min for 10 minutes using a laboratory centrifuge EBA20 (HettichLab, Germany). Then part of the platelet-poor plasma (PPP) and the “buffy coat” (the layer rich in platelets and white blood cells) were transferred to a new sterile tube and centrifuged at 1900 rpm/min for another five minutes. Part of the PPP and the buffy coat (3 ml in total) were transferred to another sterile tube where 1 ml calcium gluconate (activator) was added. The tube was carefully shaken and left for 20 minutes until a PRP gel was formed (Figure 6).

Surgical technique

**Figure 7:**
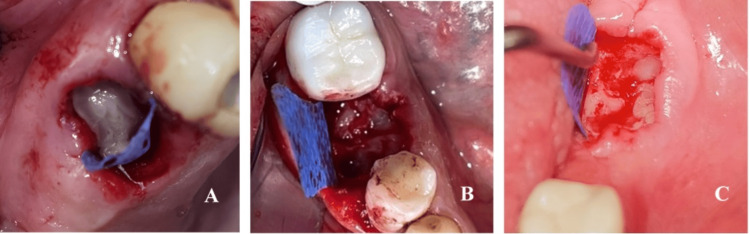
Post-extraction sockets filled with PRP (A) A premolar site; (B) and (C) a molar site. PRP: Platelet-rich plasma.

**Figure 8:**
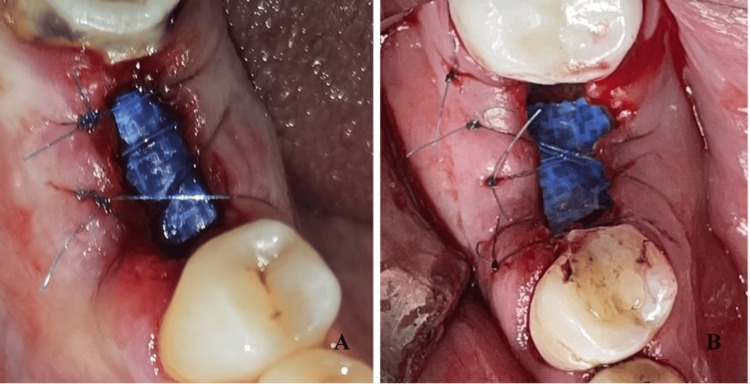
Socket sealing with a d-PTFE membrane (A, B) After membrane placement and suturing of the gingival margin. d-PTFE: Dense polytetrafluoroethylene.

Atraumatic tooth extraction was performed under infiltration anesthesia with articaine 40 mg/0.005 mg/ml. Multirooted teeth were first separated using diamond burrs with water cooling, and the roots were extracted one by one. The sockets were debrided and rinsed with saline. In the control group, the socket was sutured with crossed mattress sutures or single interrupted sutures with 5/0 non-resorbable polyamide material. In the experimental group, the gingival margin was retracted from the bone to create subperiosteal pockets for membrane insertion. The socket was then filled with the activated PRP (Figure 7). The d-PTFE membrane was shaped and inserted 3-5 mm under the periosteum covering the socket orifice and partially left exposed. The gingival margin was sutured with crossed mattress suture and/or single interrupted sutures with 5/0 polyamide (Figure 8).

Postoperative care

**Figure 9:**
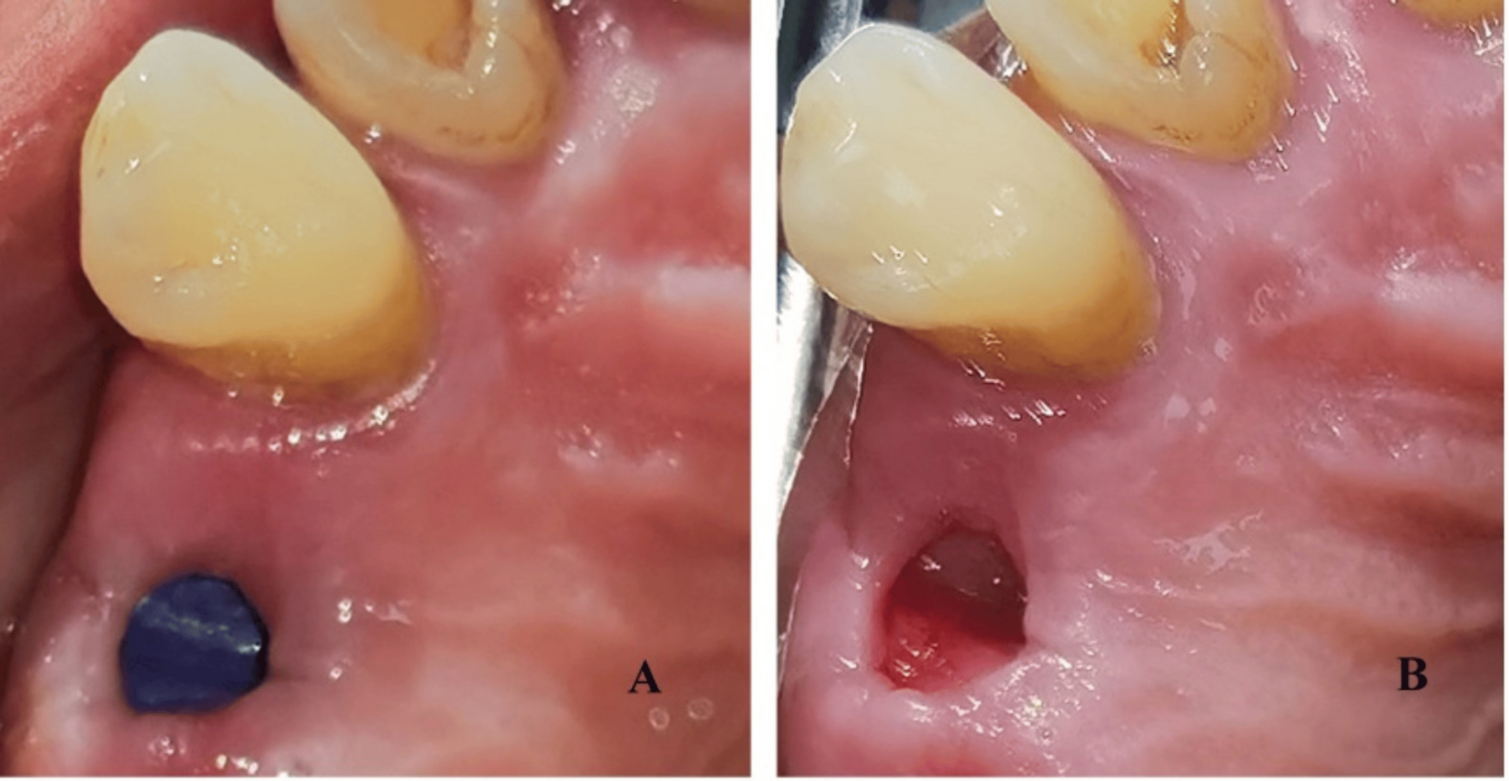
Postoperative view (A) After suture removal (day 14) and (B) after membrane removal (day 28).

Antibiotic prophylaxis was prescribed - amoxicillin with clavulanic acid 1000 mg (twice daily for seven days) or azithromycin 500 mg (one daily for five days) in case of beta-lactam allergy. Postoperative instructions for personal oral hygiene and a soft diet were given. Analgesics and mouth rinse with 0.12% chlorhexidine were also prescribed. The suture removal was done after one week in the control group and two weeks in the experimental group. The membrane removal was on the 28th postoperative day using hemostatic forceps (Figure 9). The wound epithelization after the membrane removal took two to four weeks.

Statistical analysis

Data analysis was performed using IBM SPSS v25.0 (IBM Corp., Armonk, NY), Jamovi Statistical Software, and MS Office Excel 2016 (Microsoft Corp., Redmond, WA) was used for graphical analysis. The study results were presented using descriptive statistics measures. Hypothesis testing was performed using parametric (t-test and the Pearson correlation) and non-parametric tests (Spearman correlation, Mann-Whitney U, and Pearson’s chi-squared tests).

## Results

The measurements in the experimental and control groups are presented in Table 1.

**Table 1:** Height and width of the bone plates in the experimental and control groups D-PTFE membrane: Dense polytetrafluoroethylene membrane; PRP: Platelet-rich plasma; А0: Buccal plate height on the day of the extraction; B0: Palatal/lingual plate height on the day of the extraction; Аw: Buccal plate width on the day of the extraction; Bw: Palatal/lingual plate width on the day of the extraction; А1: Buccal plate height three months after the extraction; B1: Palatal/lingual plate height three months after the extraction. * Measured in millimeters. ** The number of measurements. It exceeded the number of extracted teeth as mesial and distal measurements were taken in sockets with preserved inter-radicular septa.

Group	Measurement	N	Mean	SD	Median	IQR	Range	Min	Max
Experimental (d-PTFE membrane + PRP)	А_0_^*^	27**	13.92	4.32	13.16	4.27	8.46	6.08	24.54
	А_w_^*^		2.3	1.36	2.06	2.32	4.33	0.8	5.13
	B_0_^*^		15.35	3.45	16.46	4.42	14.58	6.23	20.81
	B_w_^*^		3.35	1.17	3.6	1.4	4.59	0.8	5.39
	А_1_^*^		12.74	4.09	12.41	3.63	19.4	5.02	24.42
	B_1_^*^		14.46	3.56	15.58	4.09	14.36	5.56	19.92
Control	А_0_^*^	24**	13.4	4.54	14.54	6.91	14.51	4.49	19
	А_w_^*^		13.54	4.63	14.8	7.34	15.35	3.88	19.23
	B_0_^*^		1.73	1.19	1.41	1.52	4.66	0.4	5.06
	B_w_^*^		2.99	1.07	2.74	1.43	4.08	1.2	5.28
	А_1_^*^		9.94	4.24	9.73	7.12	13.92	2.51	16.43
	B_1_^*^		11.46	4.51	12.05	7.61	14.89	3	17.89

**Figure 10:**
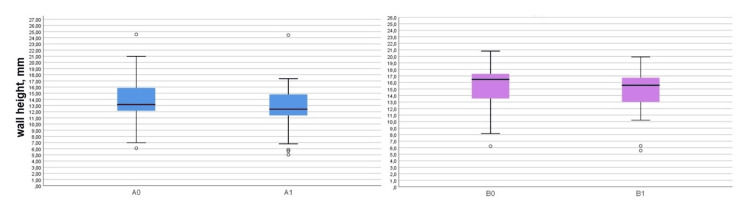
Boxplots presenting the buccal and palatal/lingual plate heights in the experimental group A0: Buccal plate height on the day of the extraction; A1: Buccal plate height three months after the extraction; B0: Palatal/lingual plate height on the day of the extraction; B1: Palatal/lingual plate height three months after the extraction.

**Figure 11:**
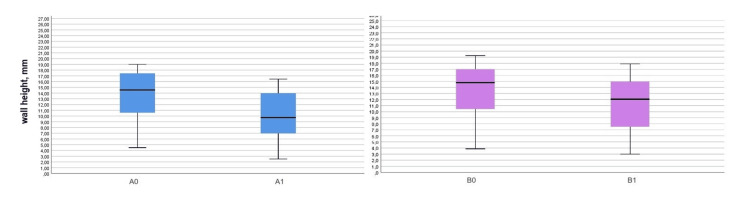
Boxplots presenting the buccal and palatal/lingual plate heights in the control group A0: Buccal plate height on the day of the extraction; A1: Buccal plate height three months after the extraction; B0: Palatal/lingual plate height on the day of the extraction; B1: Palatal/lingual plate height three months after the extraction.

Statistically significant vertical resorption of both plates was found in the experimental group as 1.18 ± 1.28 mm for the buccal plate (t = 4.79, p < 0.0001) and 0.88 ± 0.56 mm for the palatal/lingual plate (t = 8.17, p < 0.0001). The vertical plate resorption in the control group was also statistically significant: 3.47 ± 1.96 mm for the buccal plate (t = 8.66, p < 0.0001) and 2.08 ± 1.10 mm for the palatal/lingual plate (t = 9.27, p < 0.0001), as shown in Table 2. The measured plate heights are graphically presented in Figures 10, 11.

**Table 2:** Buccal and palatal/lingual plate heights on the extraction day and three months after the extraction in the experimental and control groups *A0-A1; ** B0-B1.

Group	Measurement	N	Mean	SD	Mean difference	95% CI		t	P-value
						Lower	Upper		
Experimental	A_0_	27	13.92	4.32	1.18*	0.67	1.69	4.79	<0.0001
	A_1_		12.74	4.09					
	B_0_	27	15.35	3.45	0.88**	0.66	1.11	8.17	<0.0001
	B_1_		14.46	3.56					
Control	A_0_	24	13.4	4.54	3.47*	2.64	4.29	8.66	<0.0001
	A_1_		9.94	4.24					
	B_0_	24	13.54	4.63	2.08**	1.62	2.55	9.27	<0.0001
	B_1_		11.46	4.51					

**Figure 12:**
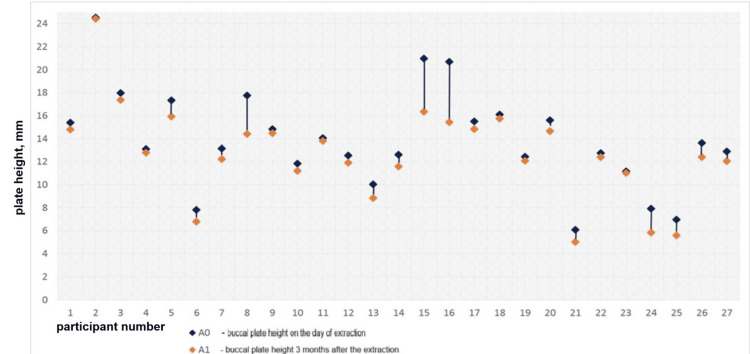
Vertical loss of the buccal plate in the experimental group for three months

**Figure 13:**
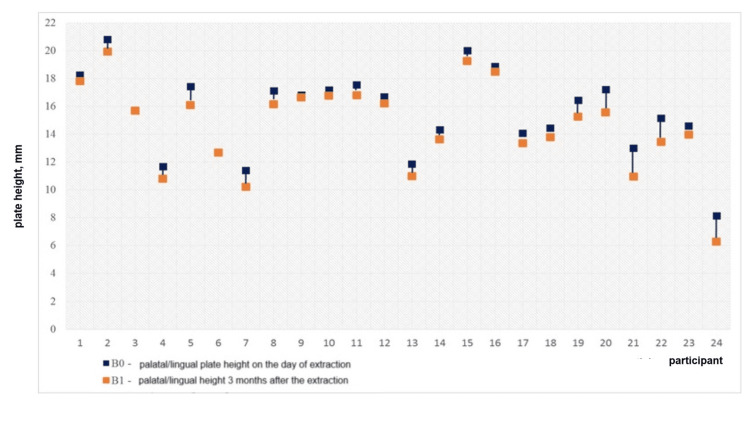
Vertical loss of the palatal/lingual plate in the experimental group for three months

The vertical bone loss in the experimental group is displayed in Figures 12, 13. The diagrams demonstrate that the difference between the two values was negligibly small in most cases, i.e., the bone resorption in the group was minimal.

**Figure 14:**
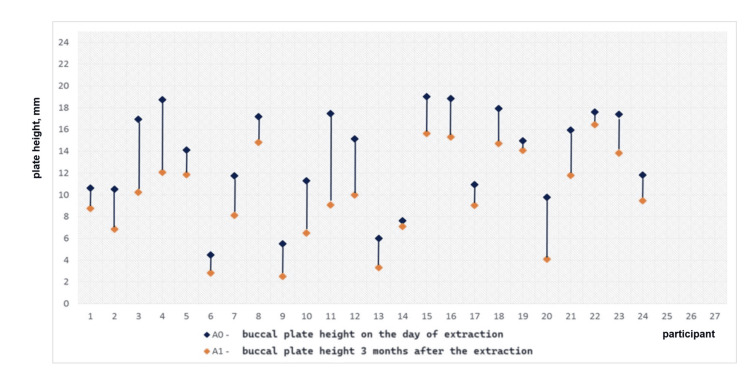
Vertical loss of the buccal plate in the control group for three months

**Figure 15:**
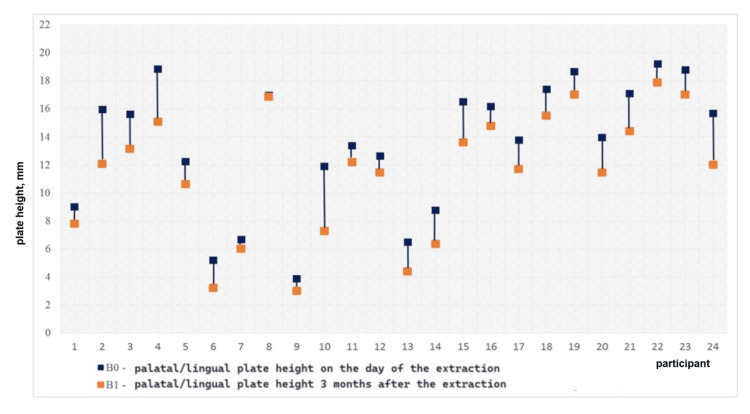
Vertical loss of the palatal/lingual plate in the control group for three months

The vertical bone loss in the control group is graphically presented in Figures 14, 15. The diagrams demonstrate pronounced vertical resorption of the plates in the control group.

The mean difference (0.3 mm) between the vertical loss of the plates in the experimental group was statistically insignificant (Mann-Whitney = 358.000, p = 0.917). A weak and statistically insignificant correlation was found between the vertical resorption of the plates (Spearman's r = 0.20, p = 0.322).

**Figure 16:**
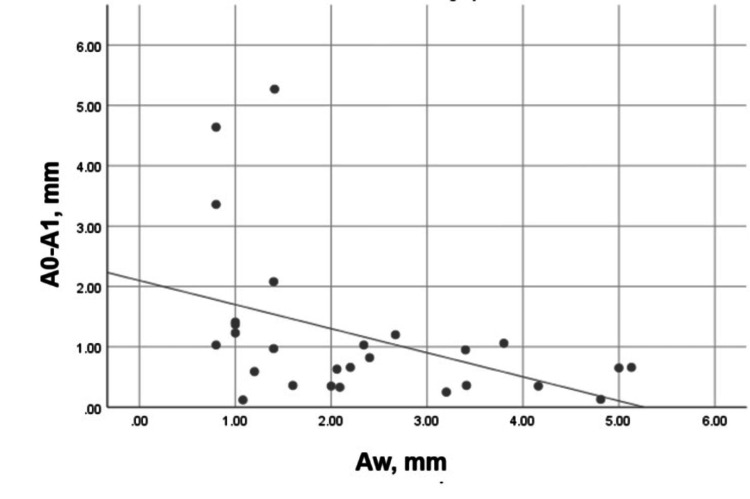
Correlation between Aw and A0-A1 in the experimental group Aw: Buccal plate width on the day of the extraction; A0-A1: Vertical loss of the buccal plate for three months.

**Figure 17:**
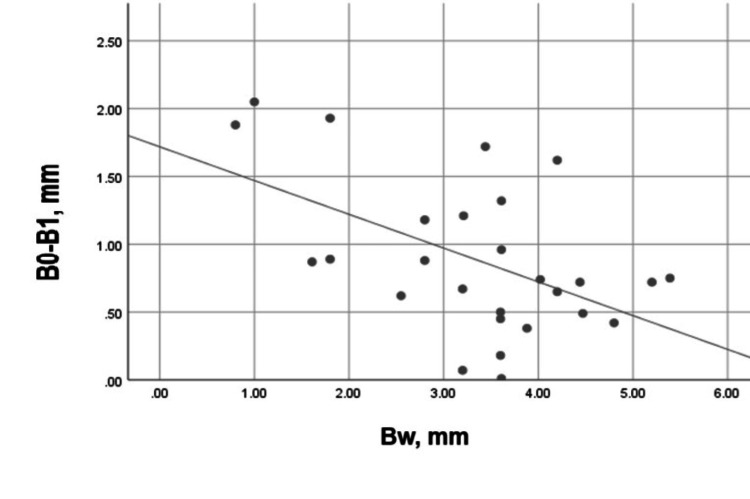
Correlation between Bw and B0-B1 in the experimental group Bw: Palatal/lingual plate width on the day of the extraction; B0-B1: Vertical loss of the palatal/lingual plate for three months.

A moderate, negative, and significant correlation (Spearman’s r = -0.487, p = 0.010) was registered between the width of the buccal plate and its vertical resorption for three months (Figure 16). This correlation was strong, negative, and statistically significant (Pearson’s r = -0.520, p = 0.005) for the palatal/lingual bone plate (Figure 17).

The mean difference (1.39 mm) between the vertical loss of the buccal and the palatal/lingual plate in the control group was statistically significant (t = 3.01, p = 0.005). The correlation between the vertical resorption of the plates was weak and statistically insignificant (Pearson’s r = 0.21, p = 0.333).

**Figure 18:**
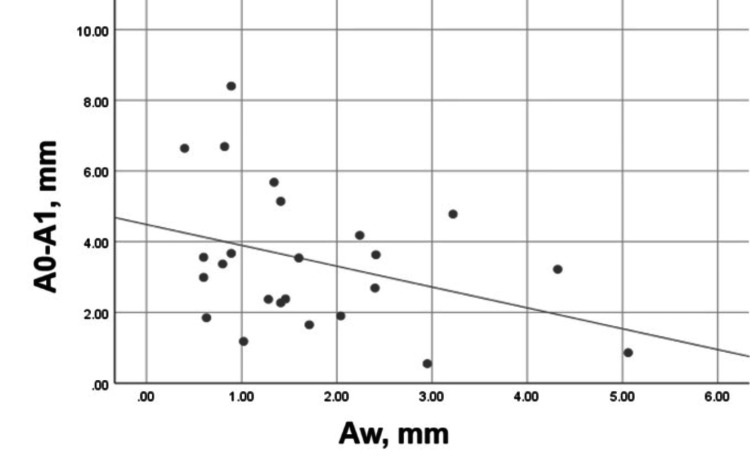
Correlation between Aw and A0-A1 in the control group Aw: Buccal plate width on the day of the extraction; A0-A1: Vertical loss of the buccal plate for three months.

A moderate, negative, and statistically insignificant correlation (Pearson’s r = - 0.358, p = 0.086) was established between the width of the buccal plate and its vertical bone resorption for three months (Figure 18).

**Figure 19:**
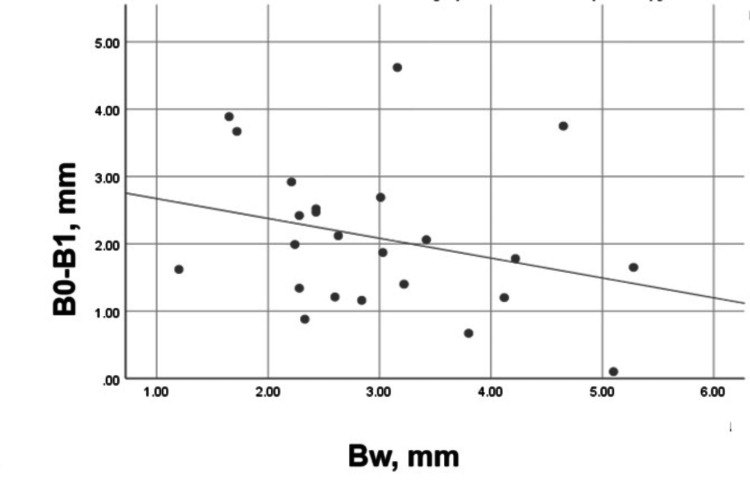
Correlation between Bw and B0-B1 in the control group Bw: Palatal/lingual plate width on the day of the extraction; B0-B1: Vertical loss of the palatal/lingual plate for three months.

The tendency was the same for the palatal/lingual bone plate but with a weak, negative, and statistically insignificant correlation (Pearson’s r = -0.287, p = 0.175), as shown in Figure 19.

**Figure 20:**
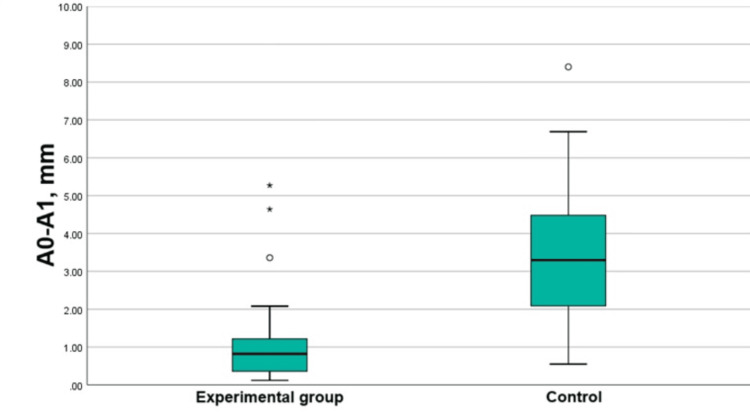
Boxplots presenting the vertical resorption of the buccal plates in the groups A0-A1: Vertical loss of the buccal plate for three months.

The vertical resorption of the buccal plate was more pronounced in the control group (Mann-Whitney = 564.000, p < 0.0001 resorption group), as shown in Figure 20.

**Figure 21:**
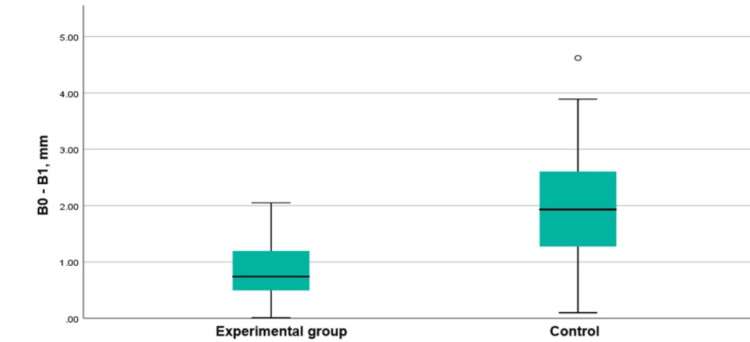
Boxplots presenting the vertical resorption of the palatal/lingual plates in the groups B0-B1: Vertical loss of the palatal/lingual plate for three months.

The comparison between the vertical resorption of the palatal plate in the experimental and control groups demonstrated a mean difference of 1.20 mm (t = -4.81, p < 0.0001). The resorption was more pronounced in the control group (Figure 21).

There was a significant difference in the vertical resorption of both thin (Aw ≤2 mm) and thick (Aw >2 mm) buccal plates between the groups (Tables 3, 4).

**Table 3:** Vertical bone loss of the thin (≤2 mm) buccal plates in the groups A0-A1: Vertical loss of the buccal plate for three months; Aw: Buccal plate width on the day of the extraction.

A_0_-A_1 _in plates with Aw ≤ 2 mm	Group	N	Mean	SD	Median	IQR	Range	Min	Max	Mann-Whitney U test	P
	Experimental	13	1.75	1.66	1.23	1.49	5.15	0.12	5.27	172	0.002
	Control	16	3.84	2.09	3.46	3.09	7.22	1.18	8.4		

**Table 4:** Vertical bone loss of the thick (>2 mm) buccal plates in the groups A0-A1: Vertical loss of the buccal plate for three months; Aw: Buccal plate width on the day of the extraction. *((A0-A1) in the control group - (A0-A1) in the experimental group).

A_0_-A_1 _in plates with A_w _>2 mm	Group	N	Mean	SD	Mean difference*	95% CI		t	P
						Lower	Upper		
	Experimental	14	0.65	0.33	-2.08	-3.36	-0.8	-3.8	0.006
	Control	8	2.73	1.53					

## Discussion

Tooth extraction leads to the resorption of the alveolar bone and clinically visible dimensional alterations. Up to 50% of the ridge width at the premolar and molar sites is resorbed during the first year after the extraction [17].

Recently, the regenerative properties of APC have been the subject of discussion among researchers in the field. Most studies on the use of PRP for SP have investigated its combined application with bone grafts. In contrast, the present research evaluates its application only in combination with a barrier membrane.

The results in the control group demonstrated that the vertical resorption of both plates was of high statistical and clinical significance. It was 3.47 ± 1.96 mm for the buccal plate and 2.08 ± 1.10 mm for the palatal/lingual plate. The difference between the vertical resorption of the two plates in the control group (mean difference: 1.39 mm) was statistically and clinically significant. It demonstrates that the resorption in the control group did not proceed evenly but was more pronounced on the buccal side.

This difference in the resorption of the plates corresponds to the results by Araújo and Lindhe. They described the volumetric changes of the ridge during the first two months of premolar extractions in the lower jaw. The increased osteoclast activity during this period led to the resorption of the socket plates, especially the buccal one. The histological results demonstrated that the margins of the buccal wall were made of bundle bone, while the lingual wall was composed of both bundle and supporting bone [18].

The vertical bone loss of the plates in the experimental group (PRP and d-PTFE membranes) was also of high statistical and clinical significance (1.18 ± 1.28 mm for the buccal plate and 0.88 ± 0.56 mm for the palatal/lingual plate). It was more pronounced in the buccal plate but without a significant difference (with a mean difference of 0.3 mm).

A weak and statistically insignificant correlation was found between the buccal and the palatal/lingual plate resorption in the control group, i.e., the plates did not affect each other. The mean difference between the buccal plate width and the palatal/lingual plate width was 1.26 mm. Furthermore, the mean buccal plate width was less than 2 mm, while that of the palatal/lingual was more than 2 mm.

A buccal plate width of less than 2 mm has been related to more pronounced bone resorption [19,20]. The exact width threshold has been debated and not yet established. Chappuis et al. found a mean vertical resorption of 7.5 mm for eight weeks in buccal plates with a width of ≤1 mm. At sites with thicker buccal walls (>1 mm), they registered a mean vertical loss of 1.1 mm [21].

A weak, negative, and statistically insignificant correlation was found between the plate widths, measured immediately on the extraction day, and the vertical resorption of the plates for three months. Therefore, the plate width was not the only determining factor for the pronounced vertical resorption in the control group. Although it was more evident for the buccal plate, the vertical resorption of both plates was of high clinical and statistical significance.

Walker et al. reported vertical resorption of the buccal plate of 2.60 ± 2.06 mm at the molar areas after three months of healing [17]. The vertical resorption reported by Barone et al. was 2.05 ± 0.72 mm (buccal wall) and 2.00 ± 0.69 mm (oral wall) [22].

A weak and statistically insignificant correlation was found between the buccal and the palatal/lingual plate resorption in the experimental group, i.e., the plates did not influence each other. The correlation between the buccal plate width and the plate vertical resorption for three months was moderate, negative, and statistically significant. For the palatal/lingual bone plate, this correlation was strong, negative, and statistically significant. This demonstrated that the "width" factor influenced the vertical resorption of both plates.

A non-linear correlation between the buccal plate width and the alveolar ridge resorption has been reported. The correlation was twice as pronounced at sites with a buccal bone width < 2 mm, i.e., the mean vertical resorption was significantly greater in these cases [19]. There was marked vertical resorption of the buccal plate in the control group, but since the mean plate width was 1.73 ± 1.19 mm (<2 mm), the "width" factor will be considered in more detail.

Some authors indicate the threshold of 2 mm as a "critical width value" [19,20], while others state that this "threshold" is 1 mm. For values ​​≤ 1 mm, the expected vertical resorption is twice [23] or three times [24] more pronounced. Moreover, this value may be affected by the application of SP.

SP with non-resorbable bone substitutes has been recommended to compensate for bone resorption in sockets with thin buccal plates (≤1 mm) [24,25]. Different barrier membranes and bone substitute materials have been utilized in guided bone regeneration [26,27]. Their application for SP should be further investigated. It has been suggested that the buccal plate width can be used as a prognostic marker for predicting bone resorption before tooth extraction [28,29].

SP procedures can reduce physiological bone resorption and promote delayed implant placement. They can limit but not eliminate the resorption process. De Angelis et al. found that the need for additional augmentation procedures was 26% in spontaneous healing and only 9% after SP [30].

Limitations

A sufficient limitation of this trial is the short follow-up period (three months), which should be extended for cases of delayed and late implant placement. Another limitation is that only the width threshold of 2 mm was assessed, instead of evaluating both “critical” widths of 1 and 2 mm. The latter is due to the limited cases of buccal plate width ≤ 1 mm.

Future directions

Further randomized controlled trials should observe the application of d-PTFE membranes and PRP (with and without bone grafts) for SP. In addition, the critical threshold (if any) in the plate width should be further investigated, along with the additional factors that can influence post-extraction ridge resorption.

## Conclusions

SP involves different techniques, such as atraumatic tooth extraction, guided regeneration with barrier membranes and bone substitutes, socket sealing, and immediate implant placement. The SP method with non-porous d-PTFE membranes and PRP, discussed in this research, can reduce vertical bone loss at the premolar and molar sites. Data analysis suggested that the buccal plate width influences the amount of its vertical resorption, but not as much as the management of the post-extraction socket (SP versus spontaneous socket healing). Further longitudinal randomized controlled trials are necessary to clarify which factors influence post-extraction ridge resorption and evaluate the success of different SP techniques.
